# Tomatidine and Patchouli Alcohol as Inhibitors of SARS-CoV-2 Enzymes (3CLpro, PLpro and NSP15) by Molecular Docking and Molecular Dynamics Simulations

**DOI:** 10.3390/ijms221910693

**Published:** 2021-10-02

**Authors:** Rafat Zrieq, Iqrar Ahmad, Mejdi Snoussi, Emira Noumi, Marcello Iriti, Fahad D. Algahtani, Harun Patel, Mohd Saeed, Munazzah Tasleem, Shadi Sulaiman, Kaïss Aouadi, Adel Kadri

**Affiliations:** 1 Department of Public Health, College of Public Health and Health Informatics, University of Ha’il, Ha’il 81451, Saudi Arabia; r.zrieq@uoh.edu.sa (R.Z.); dr.algahtani@gmail.com (F.D.A.); 2 Division of Computer Aided Drug Design, Department of Pharmaceutical Chemistry, R. C. Patel Institute of Pharmaceutical Education and Research, Shirpur, Maharashtra 425405, India; ansariiqrar50@gmail.com (I.A.); hpatel_38@yahoo.com (H.P.); 3 Department of Biology, College of Science, University of Ha’il City, P.O. 2440, Ha’il 2440, Saudi Arabia; emira_noumi@yahoo.fr (E.N.); mo.saeed@uoh.edu.sa (M.S.); 4 Laboratory of Genetics, Biodiversity and Valorization of Bio-Resources (LR11ES41), University of Monastir, Higher Institute of Biotechnology of Monastir, Avenue Tahar Haddad, BP74, Monastir 5000, Tunisia; 5 Laboratory of Bioresources: Integrative Biology and Valorization, (LR14-ES06), University of Monastir, Higher Institute of Biotechnology of Monastir, Avenue Tahar Haddad, BP74, Monastir 5000, Tunisia; 6 Department of Agricultural and Environmental Sciences, Università degli Studi di Milano, 20133 Milano, Italy; 7 Phytochem Lab., Department of Agricultural and Environmental Sciences, Università degli Studi di Milano, 20133 Milano, Italy; 8 National Interuniversity Consortium of Materials Science and Technology (INSTM), 50121 Firenze, Italy; 9 BAT Center—Interuniversity Center for Studies on Bioispired Agro-Environmental Technology, University of Napoli “Federico II”, Portici, 80055 Napoli, Italy; 10 Molecular Diagnostic and Personalized Therapeutics Unit, University of Ha’il, Ha’il 81451, Saudi Arabia; 11 School of Electronic Science and Engineering, University of Electronic Science and Technology of China, Chengdu 610054, China; munazzah.t@gmail.com; 12 Department of Clinical Laboratory Sciences, Faculty of Applied Medical Sciences, University of Ha’il, Ha’il 81451, Saudi Arabia; s.suleiman@uoh.edu.sa; 13 Department of Chemistry, College of Science, Qassim University, Buraidah 51452, Saudi Arabia; K.AOUADI@qu.edu.sa; 14 Faculty of Science of Monastir, University of Monastir, Avenue of the Environment, Monastir 5019, Tunisia; 15 Department of Chemistry, Faculty of Science and Arts of Baljurashi, Albaha University, Al Bahah 65731, Saudi Arabia; lukadel@yahoo.fr; 16 Faculty of Science of Sfax, Department of Chemistry, University of Sfax, B.P. 1171, Sfax 3000, Tunisia

**Keywords:** tomatidine, patchouli alcohol, COVID-19, drug repurposing, docking study, dynamic simulation, ADMET

## Abstract

Considering the current dramatic and fatal situation due to the high spreading of SARS-CoV-2 infection, there is an urgent unmet medical need to identify novel and effective approaches for prevention and treatment of Coronavirus disease (COVID 19) by re-evaluating and repurposing of known drugs. For this, tomatidine and patchouli alcohol have been selected as potential drugs for combating the virus. The hit compounds were subsequently docked into the active site and molecular docking analyses revealed that both drugs can bind the active site of SARS-CoV-2 3CLpro, PLpro, NSP15, COX-2 and PLA2 targets with a number of important binding interactions. To further validate the interactions of promising compound tomatidine, Molecular dynamics study of 100 ns was carried out towards 3CLpro, NSP15 and COX-2. This indicated that the protein-ligand complex was stable throughout the simulation period, and minimal backbone fluctuations have ensued in the system. Post dynamic MM-GBSA analysis of molecular dynamics data showed promising mean binding free energy 47.4633 ± 9.28, 51.8064 ± 8.91 and 54.8918 ± 7.55 kcal/mol, respectively. Likewise, in silico ADMET studies of the selected ligands showed excellent pharmacokinetic properties with good absorption, bioavailability and devoid of toxicity. Therefore, patchouli alcohol and especially, tomatidine may provide prospect treatment options against SARS-CoV-2 infection by potentially inhibiting virus duplication though more research is guaranteed and secured.

## 1. Introduction

The current contagious pandemic of the novel severe acute respiratory syndrome coronavirus 2 named SARS-CoV-2, is a viral pneumonia and a threat to global public health [1,2]. According to the current situational report (https://www.worldometers.info/coronavirus/), released on 22 July 2021, the SARS-CoV-2 has already affected over 179 million people across the world, including 396,758 confirmed cases in Kingdom Saudi Arabia with 7691 deaths. The SARS-CoV2 is a single-stranded positive-sense RNA genome of size 29.7 kb [3]. Structurally, it was composed of Spike glycoprotein (S), Envelope protein (E), Membrane protein (M) and Nucleocapsid protein (N). Envelope (E) protein is essential to release the virus, Membrane (M) protein by increasing the membrane curvature, promotes the viral assembly; Nucleocapsid (N) protein is interferon (IFN) antagonistic and supports viral replication. Hemagglutinin esterase (HE) and Helicase (H) proteins, and nonstructural proteins (NSP) which include proteases, papain-like proteases (PL^pro^ or NSP3) and 3C-like protease (M^pro^ or NSP5), and Replicase proteins [4,5,6]. The envelope and nucleocapsid proteins of SARS-CoV-2 are two evolutionarily conserved regions, with sequence identities of 96% and 89.6%, respectively [7]. M^pro^ and PL^pro^ play significant roles in the post-translational modification of the two replicase polyproteins, pp1a and pp1ab [8,9]. M^pro^ display a central role in proteolysis, viral replication processes, transcriptional processes and is essential for the life cycle of the virus [10]. PL^pro^ is essential for replication/transcription complex (RTC) formation and is responsible for release of NSP1, NSP2 and NSP3 from the N-terminal region of pp1a and 1ab [11]. Their inhibition can block these processes and prevent the viral infection. NSP15 protein also acts as an endoribonuclease and preferentially cleaves 3′ of uridylates through a ribonuclease A (RNase A)-like mechanism and also facilitates viral replication and transcription [10]. As a part of the body’s immune response, inflammation is a crucial parameter that may be taken into account to reduce infection, limit viral replication and transmission. NF-κB-inducing kinase (NIK), Cyclooxygenase-2 (COX-2), phospholipase A2 (PLA2) and interleukin-1 receptor associated kinase 4 (IRAK-4) are important druggable targets involved in SARS-CoV-2 induced inflammatory response and can be used to screen anti-inflammatory molecules. In fact, NIK activates NF-κB2 by promoting proteolytic processing and the generation of NF-κB transcription of the targeted gene, also known to regulates both inflammation-induced and tumor-associated angiogenesis [12]. COX-2 catalyze the biosynthesis of prostaglandins, prostacylins and thromboxanes, from arachidonic acid mostly involved in pathological conditions such as inflammation, pain and fever [11]. PLA2 enzymes are required to increase the level of arachidonic acid for metabolism and biosynthesis of eicosanoid under physiological condition as well as in inflammatory cell activation [11]. Interleukin-1 receptor-associated kinase 4 (IRAK-4) plays a pivotal role in signaling cascades associated with the immune and inflammatory diseases, and may be an effective therapeutic target for various diseases associated with deregulated inflammation [11].

Alongside the development of new, often high-budget drugs, different strategies were applied in parallel with drug repurposing which remains the best approach to rapidly identify antiviral plausible therapeutic agents. Accordingly, computational approaches to identify suitable and adequate therapeutic options in the short-term and loss-cost, to explore possible interactions with viral proteins have been well investigated [13,14]. The molecular docking and dynamic simulation process were largely applied in structure-based drug design owing to their ability to predict, with reliable accuracy which are intended to decipher the mechanism of binding interactions [15,16].

Extensive research on the therapeutic effect of traditional medicinal plant remedies, have been highlighted against various health ailments as well as an alternative medicine [13,17,18,19,20]. A particular attention was offered a phytocomponents showing scientific evidence for host protection against SARS-CoV-2 infections. Their effectiveness effect is employed in strengthening of host immune system with less adverse side effects, in contrast with synthetically generated chemical substances to cure many diseases.

Tomatidine as a steroidal alkaloid widely found in skins and leaves of eggplant, potatoes and tomatoes [21]. This steroidal was reported to inhibit the replication of *Staphylococcus aureus* as well as the invasion activity of A549 human lung adenocarcinoma cells and induces HL60 human myeloid leukemia cell apoptosis [22,23,24]. In addition, tomatidine suppresses iNOS and COX-2 expression by blocking the NF-κB pathway in LPS stimulated RAW 264.7 cells [25]. Recently tomatidine was proved to possess anti-inflammatory effects in macrophages [26]. Other study reported that tomatidine exhibited antiviral activity towards chikungunya virus [27]. Patchouli alcohol, as naturally occurring tricyclic sesquiterpene, has been widely demonstrated for its multi-beneficial pharmacological properties such as immunomodulatory, antitumor, anti-inflammatory, antimicrobial, antioxidative, insecticidal, antiatherogenic and antiemetic [28].

Repurposing existing drugs offers the fastest opportunity to identify new indications for existing drugs as a stable solution against coronavirus disease 2019 (COVID-19). Since, an effective drug against SARS-CoV-2 still needs to be discovered, in this study, we recruited an amalgam of docking-based virtual screening, molecular dynamics (MD) simulations and binding-free energy approaches to identify suitable existing drugs for the treatment of COVID-19. We believe that our findings will help pharmaceutical chemists to optimize suitable drugs for the clinical management of COVID-19 patients. Therefore, based on the above facts, in this study, we performed the molecular docking and dynamic simulation of tomatidine and patchouli alcohol in order to discover a novel potential inhibitor to 3CL^pro^, PL^pro^ and NSP15 as well as pro-inflammatory mediators such as COX-2 and PLA-2 enzymes. Additionally, pharmacokinetics, drug-likeness and target prediction were assessed.

## 2. Results and Discussion

### 2.1. Molecular Docking Analysis

Patchouli alcohol is one of the important compounds isolated from the essential oil of Pogostemon cablin (patchouli), in parallel with tomatidine as a glycoalkaloid and an agly-cone metabolite of tomatidine which is largely present in high amount in unripe tomato and were tested here, for the first time for their potential effect as anti-SARS-CoV-2, using in silico methods. Proteases 3CLpro and PLpro were targeted to virtually identify the potential drug specifically blocking its catalytic dyad and triad, respectively. Their inhibition plays a pivotal role either in stopping the production of functional SARS-CoV-2 polypeptides or in reducing the viral replication on the host.

The docking method involves searching for binding sites on the whole macromolecular surface. Therefore, (SARS-CoV-2) inhibitors were used for blind docking analysis of the known drugs, tomatidine and patchouli alcohol. The protein-ligand interactions of the stable docked complexes were investigated. According to the docking score and binding mode analyses, both leads were found to tightly bind to the different selected targets.

#### 2.1.1. Docking Analysis of Tomatidine and Patchouli Alcohol with Target 3CLpro

**Figure 1:**
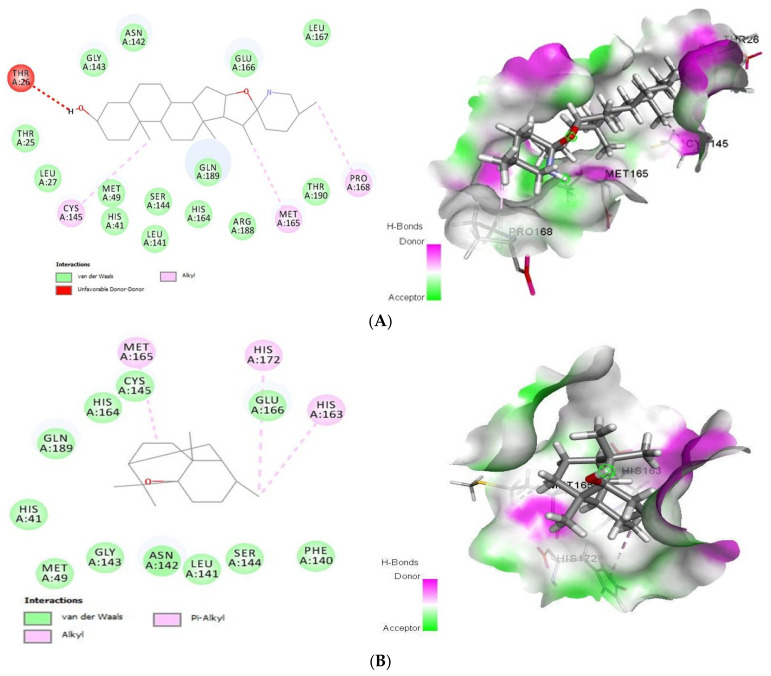
2D (left) and 3D (right) of tomatidine (**A**) and patchouli alcohol (**B**) with the active site of 6LU7. (**A**): Tomatidine-3CL-protease (PDB ID: 6LU7; (Gold Fitness Score 41.42). Type of the binding: van der Waals: Thr25, Leu27, Met49, His41, Ser144, Leu141, His164, Gln189, Arg188, Thr190, Glu166, Gly143, Asn142, Leu167; Unfavorable donor-donor: Thr26; Alkyl: Cys145, Met165, Pro168. (**B**): Patchouli alcohol-3CL-protease (PDB ID: 6LU7); (Gold Fitness Score 30.65). Type of the binding: van der Waals: His41, Met49, Gly143, Asn142, Leu141, Ser144, Phe140, Cys145, His164, Glu166, Gln189; Alkyl: Met165, His172, His163.

As shown (Figure 1), tomatidine interacts to 3CLpro (PDB ID: 6LU7) via van der Waals interactions with Thr25, Leu27, His41, Met49, Leu141, Asn142, Gly143, Ser144, His164, Glu166, Leu167, Arg188, Gln189 and Thr190, unfavorable donor-donor interactions with Thr26 and Alkyl interactions with Cys145, Met165 and Pro168, amino-acids, however patchouli alcohol established van der Waals interactions with His41, Met49, Phe140, Leu141, Asn142, Gly143, Ser144, Cys145, His164, Glu166 and Gln189, and Alkyl interactions with His163, Met165 and His172 residues. In such a case, all the residues involved in the interaction sites of the tomatidine-3CLpro complex, and patchouli alcohol-3CLpro are located in the preferred high-volume pocket of 3CLpro having Thr24, Thr25, Thr26, Leu27, His41, Cys44, Thr45, Ser46, Met49, Pro52, Tyr54, Phe140, Leu141, Asn142, Gly143, Ser144, Cys145, His163, His164, Met165, Glu166, Leu167, Pro168, His172, Asp187, Arg188, Gln189, Thr190 and Gln192 residues. We note also the presence of both Cys145 and His41 catalytic dyad in tomatidine-3CLpro complex, and patchouli alcohol-3CLpro active center known for their major role in substrate binding and the enzyme activity.

Regarding the binding mode of tomatidine and patchouli alcohol with target 3Clpro, our results are in good agreement with what has been proven for SARS-CoV-2 NSP15 indicating that His235, Gln245, Gly248, Gln294 and Thr341 were the main residues in NSP15 active site. We note also that both tomatidine and patchouli alcohol do interact with the important catalytic residue Lys290 via van der Waals and H-bonds interactions, respectively, which is involved preferentially in the hydrolysis of a nucleoside as observed from SARS and MERS NSP15 [7]. Interestingly, the residue His235 which is pivotal for hydrolysis was found in. Additionally, hydroxyl groups of Ser294 and Tyr343 residues which are close to the catalytic residues and may be responsible for the binding of the substrate also make hydrogen bonds with ligands in the models. Moreover, in the case of MERS (NSP15), the importance of the residue Tyr343 for ribonuclease activity has been demonstrated by mutational studies [29].

#### 2.1.2. Docking Analysis of Tomatidine and Patchouli Alcohol with Target Plpro

**Figure 2:**
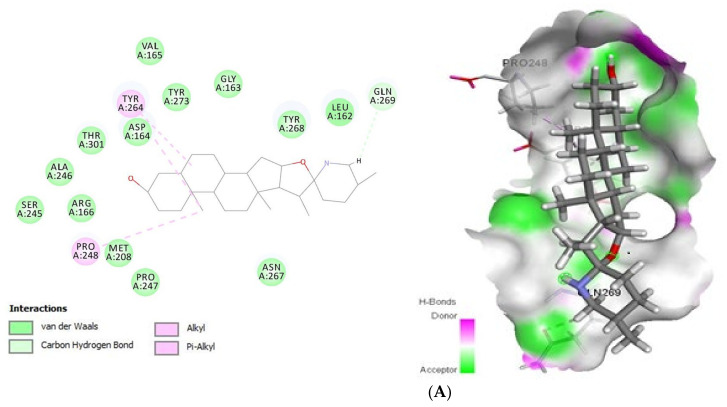

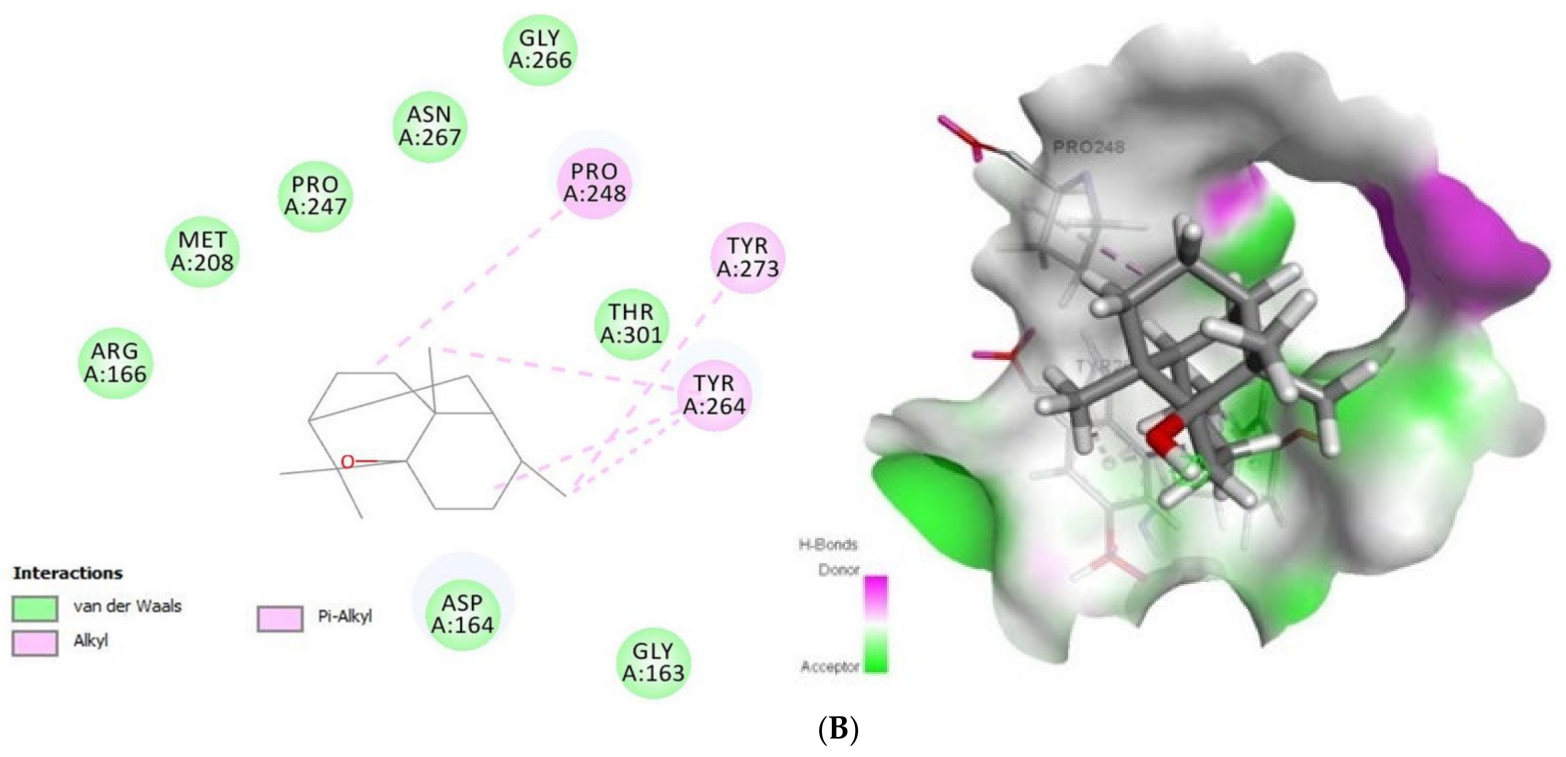
2D (left) and 3D (right) of tomatidine (**A**) and patchouli alcohol (**B**) with the active site of COVID-19 6W9C. (**A**): Tomatidine-Papain-like protease (PDB ID: 6W9C) (Gold Fitness Score37.49); Type of the binding: van der Waals: Val165, Asp164, Tyr273, Gly163, Tyr268, Leu162, Ser245, Ala246, Thr301, Asn267, Pro247, Met208, Arg166; C-H bonds: Gln269; Alkyl/Pi-Alkyl: Tyr264, Pro248. (**B**): Patchouli alcohol-Papain-like protease (PDB ID: 6W9C) (Gold Fitness Score26.44); Type of the binding: van der Waals: Asp164, Gly163, Thr301, Gly266, Asn267, Pro247, Met208, Arg166; Alkyl/Pi-Alkyl: Pro248, Tyr273, Tyr264.

**Figure 3:**
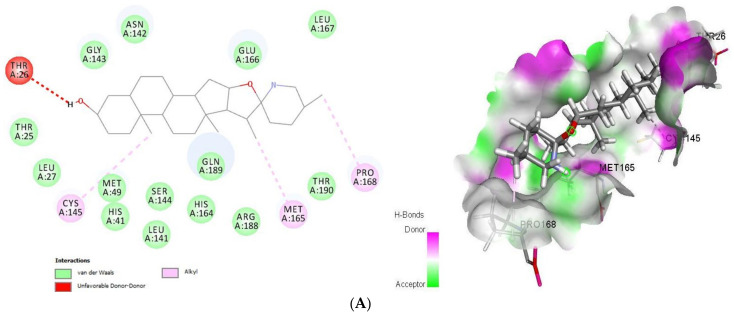

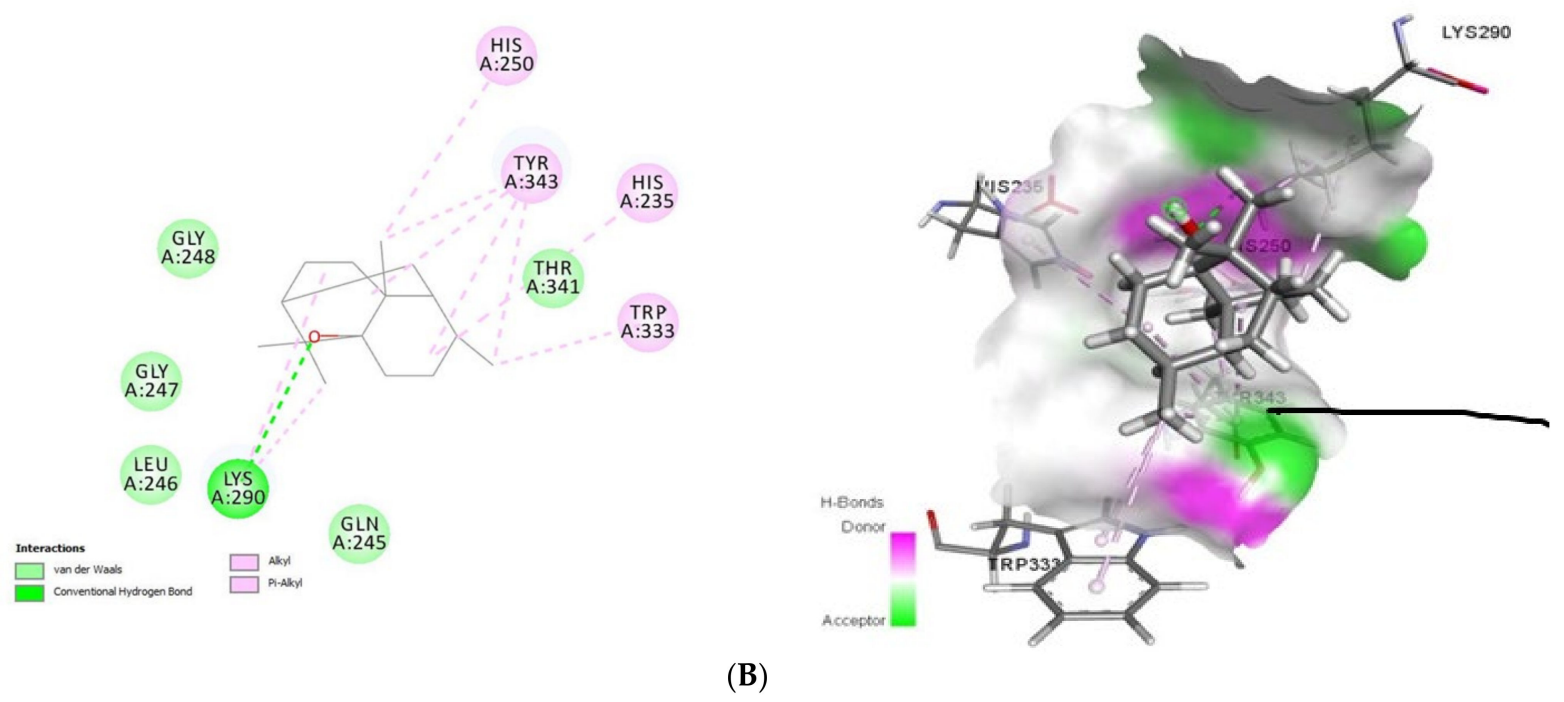
2D (left) and 3D (right) of tomatidine (**A**) and patchouli alcohol (**B**) with the active site of 6VWW. (**A**): Tomatidine-NSP15 (PDB ID: 6VWW) (Gold Fitness Score 43.62); Type of binding: van der Waals: Pro344, Lys345, Leu346, Ser294, Val292, Cys293, Gly247, Lys290, Gly248, Thr341, Lys345, Trp333, Glu340; C-H bond: His235; Pi-Sigma: Tyr343; Pi-Alkyl: His250, Tyr343, His235. (**B**): Patchouli alcohol-NSP15 (PDB ID: 6VWW) (Gold Fitness Score 27.47); Type of binding: van der Waals: Gly248, Glu247, Leu246, Gln245, Thr341; H-bond: Lys290; Alkyl/Pi-Alkyl: His250, Tyr343, His235, Trp333.

Concerning Plpro (PDB ID: 6W9C) (Figure 2) it formed van der Waals interactions with the tomatidine active site residues van der Waals: Leu162, Gly163, Asp164, Val165, Arg166, Met208, Ser245, Ala246, Pro247, Asn267, Tyr268, Tyr273, Thr301, C-H bonds with Gln269 and Alkyl/Pi-Alkyl with Pro248 and Tyr264, while patchouli alcohol established van der van der Waals: Gly163, Asp164, Arg166, Met208, Pro247, Gly266, Asn267, Thr301 and Alkyl/Pi-Alkyl with Pro248, Tyr264 and Tyr273 residues. Concerning the interaction mode of tomatidine and patchouli alcohol with target Plpro, our results were totally consistent with the Plpro S3/S4 pocket having the following residues, Asp164, Val165, Arg166, Glu167, Met 208, Ala246, Pro247, Pro248, Tyr264, Gly266, Asn267, Tyr268, Gln269, Cys217, Gly271, Tyr273, Thr301 and Asp302 confirming the potentiality of tomatidine and patchouli alcohol to inhibit the SARS-CoV-2. In the same way, docking results (Figure 3) showed that tomatidine and patchouli alcohol were well bounded at the active site of NSP15.

#### 2.1.3. Docking Analysis of Tomatidine and Patchouli Alcohol with Target NSP15

Docking results (Figure 3) showed that tomatidine exhibited interactions at the active site of NSP15 (PDB ID: 6VWW) with Gly247, Gly248, Lys290, Val292, Cys293, Ser294, Trp333, Pro344, Lys345, Leu346, Thr341, Lys345, Glu340 (van der Waals), His235 (C-H bond), Tyr343 (Pi-Sigma) and His250, Tyr343, His235 (Pi-Alkyl) residues, while patchouli-NSP15 alcohol was stabilized by the following interactions with Lys290 (H-bond) and Gln245, Leu246, Glu247, Gly248, Thr341 (van der Waals) and His250, His235, Trp333 and Tyr343 (Alkyl/Pi-Alkyl) amino acids.

#### 2.1.4. Docking Analysis of Tomatidine and Patchouli Alcohol with Anti-Inflammatory Mediators

Upon infecting the host cells, human lung epithelial cells are among the first targets for viral entry. In response to viral multiplication and host cell damage, lung epithelial cells secrete inflammatory mediators to initiate and exacerbate host innate inflammatory.

#### 2.1.5. Docking Analysis of Tomatidine and Patchouli Alcohol with Target COX-2

**Figure 4:**
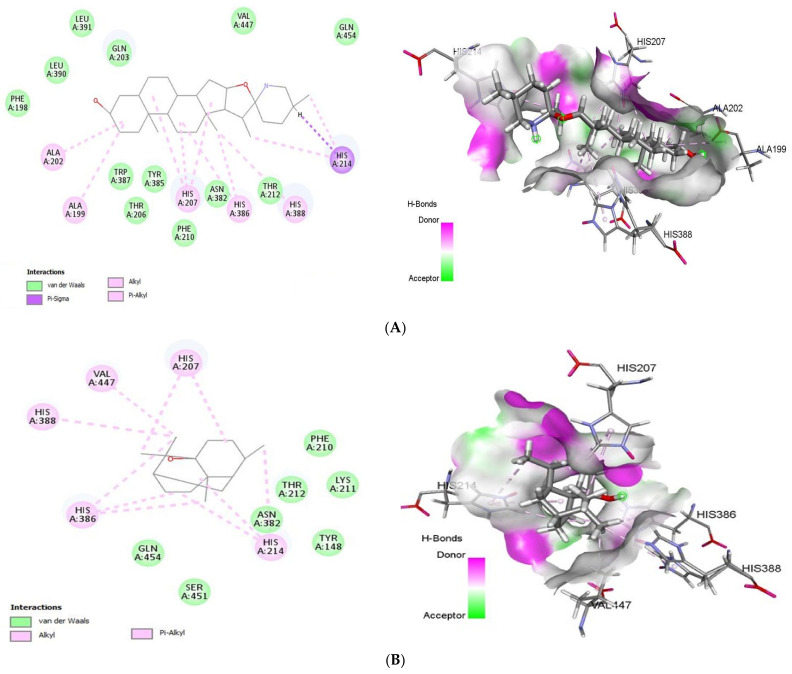
2D (left) and 3D (right) of tomatidine (**A**) and patchouli alcohol (**B**) with the active site of 5F1A. (**A**): Tomatidine-COX-2 (PDB ID: 5F1A) (Gold Fitness Score 43.64); Type of binding: van der Waals: Leu390, Leu391, Gln203, Val447, Gln454, Thr212, Asn382, Phe210, Tyr385, Thr206, Trp387, Phe198;Pi-Sigma: His214; Pi-Alkyl: His388, His386, His207, Ala199, Ala202, His214. (**B**): Patchouli alcohol-COX-2 (PDB ID: 5F1A) (Gold Fitness Score 27.73); Type of binding: van der Waals: Phe210, Lys2111, Thr212, Asn382, Tyr148, Ser451, Gln454; Alkyl/Pi-Alkyl: His214, His386, His388, Val447, His207.

COX-2 (PDB ID: 5F1A) is one of cyclooxygenase (COX) that able to catalysis the reaction forming prostaglandins and leukotrienes, respectively, from arachidonic acid, which is pathological conditions such as inflammation, pain and fever and respiratory diseases. Tomatidine bound to COX-2 with a Gold Fitness Score of 43.64 (Figure 4) establishing the van der Waals interactions with Phe198, Thr206, Phe210, Thr212, Asn382, Tyr385, Trp387, Leu390, Leu391, Gln203, Val447 and Gln454, Pi-Sigma interactions with His214 and Pi-Alkyl interactions with His388, His386, His207, Ala199, Ala202 and His214 In contrast, patchouli alcohol binds to the same target (−27.73 kcal/mol) with the residues Tyr148, Phe210, Lys211, Thr212, Asn382, Ser451, Gln454 (van der Waals) and His207, His214, His386, His388, Val447 (Alkyl/Pi-Alkyl). 

The generated active site of human COX-2 enzyme contains the following amino acids Tyr148, Ala199, Phe200, Ala202, Gln203, Thr206, His207, Phe210, Lys211, Thr212, Asp213, His214, Asn222, Val291, Gln289, Asn382, His386, Trp387, His388, Tyr389, Leu390, Leu391, Val447. As shown our results corroborate perfectly with COX-2 residues active site justifying their anti-SARS-CoV2 potency.

#### 2.1.6. Docking Analysis of Tomatidine and Patchouli Alcohol with Target sPLA-2

**Figure 5:**
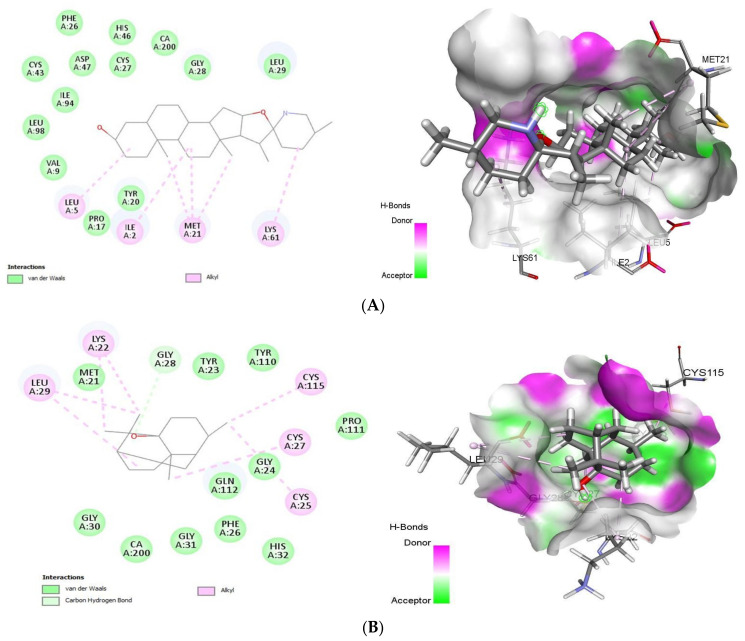
2D (left) and 3D (right) of tomatidine (**A**) and patchouli alcohol (**B**) with the active site of 4UY1. (**A**): Tomatidine-sPLA2 (PDB ID: 4UY1) (Gold Fitness Score 42.59); Type of binding: van der Waals: Val9, Leu98, Ile94, Cys43, Asp47, Phe26, His46, Cys27, Gly28, Leu29, Tyr20, Pro17; Alkyl: Leu5, Ile2, Met21, Lys61. (**B**): Patchouli alcohol-sPLA2 (PDB ID: 4UY1) (Gold Fitness Score 38.28) Type of binding: van der Waals: Met21, Tyr23, Tyr110, Pro111, Gly24, Gln112, Phe26, His32, Gly31, CA200, Gly30; C-H bond: Gly28; Alkyl: Leu29, Lys22, Cys115, Cys27, Cys25.

As summarized in Figure 5, tomatidine binds to the PLA2 **(PDB ID: 4UY1)** via hydrophobic interactions with Ile2, Leu5, Val9, Pro17, Tyr20, Met21, Phe26, Cys27, Gly28, Leu29, Cys43, His46, Asp47, Lys61, Ile94, Leu98, whereas the interaction between patchouli alcohol and PLA2 resulted in binding score of -38.28 kcal/mol, form hydrophobic interactions with Met21, Lys22, Tyr23, Gly24, Cys25, Phe26, Cys27, Gly28, Leu29, Gly30, His32, Gly31, Tyr110, Pro111, Gln112, Cys115 residues. 

With the target sPLA-2, tomatidine and patchouli alcohol interacts with Gly28 through formation of hydrogen bond which similar to several potent sPLA2-X inhibitors including varespladib, 4-benzylbenzamide derivative (AZD2716) and pyrazole derivatives [30]. In addition, our finding is similar to that of varespladib, cubebin and piperine sharing many common residues [30].

### 2.2. Molecular Dynamic Simulation and Post Dynamic MMGBSA Binding Free Energy Analysis

Since molecular docking studies were carried out using the protein rigid crystal structure, we have studied target receptor and lead compound interactions in the dynamic behavior of both receptor and ligand using molecular dynamic simulation to investigate the stability of bound conformation after binding of lead compound within the binding cavity of SARS CoV-2 3CLpro, NSP15 Endoribonuclease and human COX-2. To study the conformational stability of the Tomatidine-protein complex and its changes, we have simulated the systems up to 100 ns. In this study, we employed a period of 100 ns, which is sufficient time for the configurations of Cα atoms in complex with lead compound in 3CLpro, NSP15 and human COX-2.

The Root Mean Square Deviation (RMSD) and Root Mean Square Fluctuation (RMSF) values of the Cα atoms of proteins were calculated to indicate thermodynamic conformational stability during a 100 ns time period. The RMSD analysis indicates the simulation’s general status and whether it has equilibrated or not. It is believed that the lower the RMSD value throughout the simulation, shows higher stability of the protein-ligand complex. Conversely, greater the RMSD value, reveals the less stable the protein-ligand complex. For protein biomolecules, fluctuations in the lower RMSD range are perfectly acceptable. However, fluctuations in the wide range may indicate that the protein is likely to undergo substantial or significant conformational change during the simulation [8,9].

**Figure 6:**
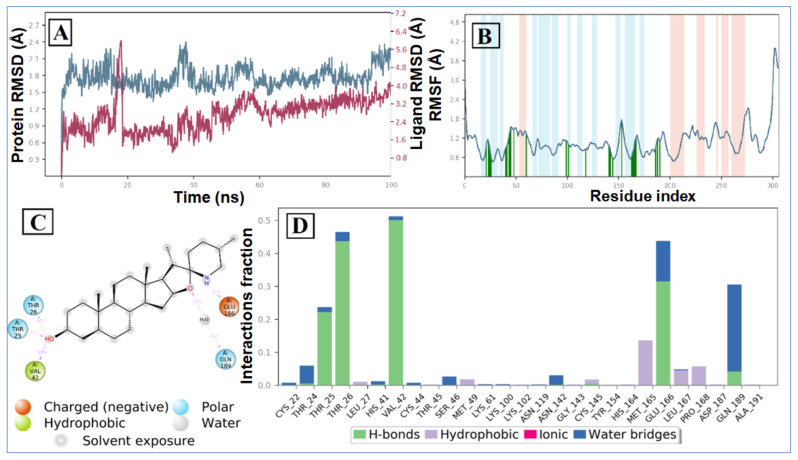
MD simulation analysis of tomatidine-3CLpro complex: (**A**) RMSD (protein RMSD is shown in grey while RMSD of tomatidine are shown in red), (**B**) protein RMSF, (**C**) 2D interaction diagram and (**D**) protein–ligand contact analysis of MD trajectory.

**Figure 7:**
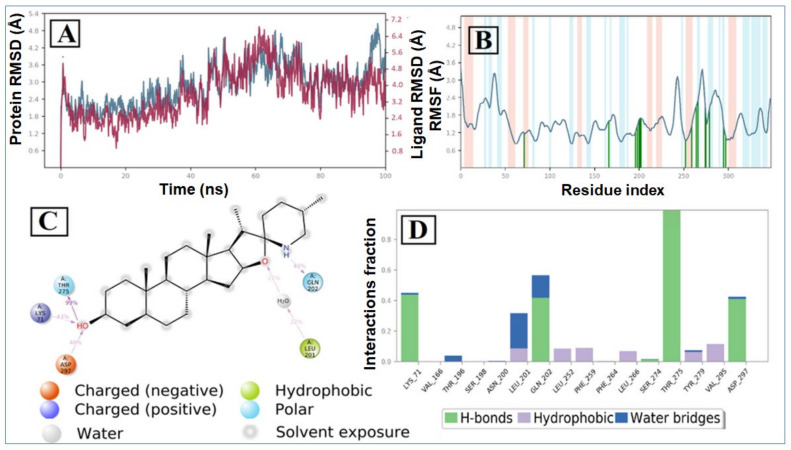
MD simulation analysis of tomatidine—SARS CoV-2 NSP15 complex: (**A**) RMSD (protein RMSD is shown in grey while RMSD of tomatidine are shown in red), (**B**) protein RMSF, (**C**) 2D interaction diagram and (**D**) protein–ligand contact analysis of MD trajectory.

The RMSD plot of the tomatidine-3CLpro complex is displayed in Figure 6A. For the RMSD study, the backbone of the protein structures enumerated throughout the MD simulation was aligned to the initial structure. After the initial fluctuation due to the equilibration, the RMSD of the tomatidine in complex with SARS CoV-2 3CLpro system gradually increased for 18ns, after which the RMSD remained between 1.2 Å to 2.5 Å till the end of the simulation. In this complex highest fluctuation up to 5.8 Å was observed at a 16–18 ns time span. Targeted protein 3CLpro revealed a maximum RMSD value of 2.7 Å, which indicates that the tomatidine-3CLpro complex was maintained constantly during the simulation time. In SARS CoV-2 NSP15- tomatidine complex, for the initial phase of the simulation showed higher fluctuations up to 5 Å because of the equilibration. For the initial 61 ns of the simulation, the tomatidine RMSD plot gradually increases, later 40 ns the tomatidine fluctuations remained within the range of 2.0 Å, indicating stabilization in protein complex structure. A similar RMSD pattern was observed with NSP15 protein as portrayed in Figure 7A.

**Figure 8:**
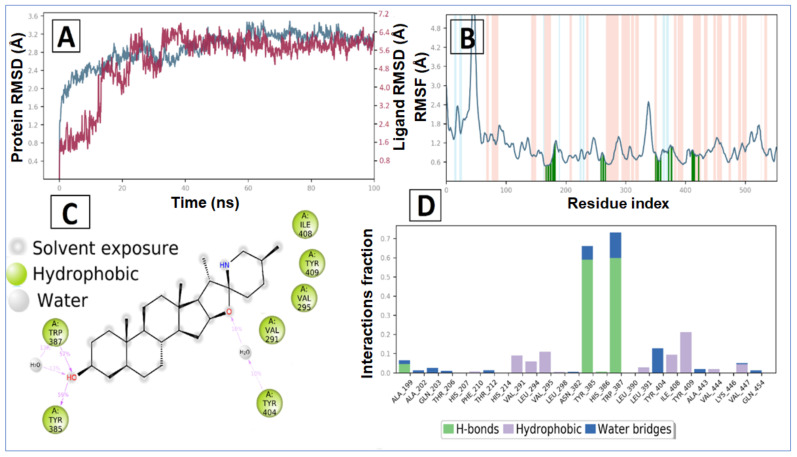
MD simulation analysis of tomatidine -human COX-2 complex: (**A**) RMSD (protein RMSD is shown in grey while RMSD of tomatidine are shown in red), (**B**) Protein RMSF, (**C**) 2D interaction diagram and (**D**) protein–ligand contact analysis of MD trajectory.

The tomatidine in complex with human COX-2 exhibited an average RMSD and RMSF value of 5.19 Å and 1.97 Å, respectively. Initially, the RMSD of the ligand increases up to 13 ns, after which a promising result was observed until 100 ns, having a constant RMSD value of 6.0 Å. In the RMSD plot of protein, initially RMSD increases up to a 23 ns time span, after that minor RMSD variation was noticed that suggests lead compound tomatidine bound tightly within the cavity of human COX-2 (Figure 8A).

The binding efficiency of lead compound tomatidine was also examined using RMSF values for Cα atoms of all the protein residues based on 100 ns trajectory data. The RMSF value reveals the mobility and flexibility of each amino acid in a protein throughout the simulation time. Larger RMSF values indicate more flexibility during simulation, while lower RMSF values indicate good system stability. In this plot, secondary structural elements, such as the α-helical and β-strand regions, are shown in red and blue backgrounds, respectively, while the loop area is shown in a white background. α-helical and β-strands are often stiffer than the unstructured portion of the protein and so fluctuate less than loop regions. If the active site and main chain atoms fluctuated little, it showed that the conformational change was minimal, suggesting that the reported lead compound was snugly bound within the cavity of the target protein binding pocket 5,6.

According to the 3CLpro RMSF plot, the RMSF value of protein backbone residues is in the range of 0.4 Å to 4.5 Å in the catalytic domain, showing high fluctuation in C- and N-terminal in contrast to other regions of the protein. The RMSF of most of the amino acid residues were within 1.6 Å and only Tyr154 show little bit higher fluctuation at 1.85 Å (Figure 1B). The residues Thr26, Thr25, Val 42 and Gln166, exhibited hydrogen bond interactions with the tomatidine. The Met165, Leu167 and Pro168 showed hydrophobic interactions during the MD simulation. The larger majority of the interactions observed between the 3CLpro and tomatidine during the docking were retained during the MD simulation, indicating a stable binding mode prediction during the docking (Figure 6C,D). The RMSF plot of tomatidine -NSP15complex is displayed in Figure 2B. The fluctuation of amino acid residues during the interaction was observed to be below 2.4 Å, which is perfectly acceptable. The highest fluctuation was identified with Val36, Asp273, Val39, Ser242, Ser242, Ile270, Asp37, Pro271, Gln347, Ser244, Gly38, His243 and Met272, which did not interact with tomatidine. The terminal hydroxyl group of tomatidine established three hydrogen bonds with Thr275, Lys71 and Asp297. The active site amino acid residues of the SARS CoV-2 NSP15 contribute to binding interactions of 99%, 43% and 40%, respectively. In tomatidine-NSP15 complex, strong hydrogen bonding was noticed with amino acid Lys71, Gln202, Thr275 and Asp297 (Figure 7C,D). 

The average RMSF measured for COX-2 upon binding of tomatidine is 1.129 Å as all the binding cavity residues fluctuated within the RMSF range of 0.6 Å to 1.7 Å, which divulges the minimum fluctuation and relative secondary conformational stability of human COX-2 upon binding of reported lead compound. The RMSF plot of tomatidine COX-2 complex is displayed in Figure 3B, where tomatidine showed interaction with Ala199, Ala202, Gln203, Thr206, His207, Phe210, Thr212, His214, Val291, Leu294, Val295, Leu298, Asn382, Tyr385, His386, Trp387, Leu390, Leu391, Tyr404, Ile408, Tyr409, Ala443, Val444, Lys446, Val447, Gln454, indicated by green vertical line. The active site amino acid residues of COX-2 protein contributing to binding interactions with tomatidine were Trp387 and Tyr385, which produced 57% and 59%, respectively (Figure 6C). Tomatidine also interacted with different COX-2 residues via hydrophobic interaction and water mediated hydrogen bonding during the simulation, as shown in Figure 8D.

**Figure 9:**
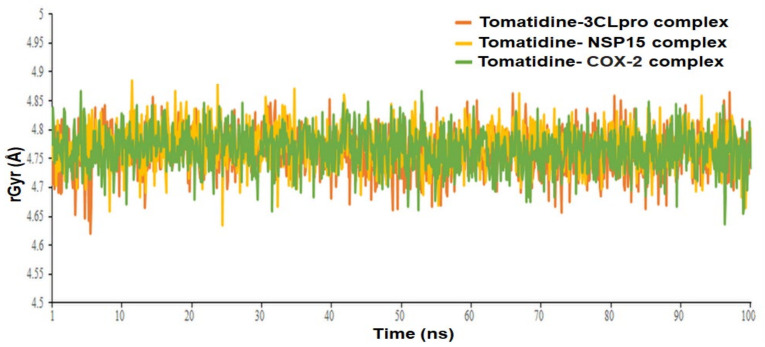
Radius of gyration (rGyr) graph of tomatidine in complex with SARS CoV-2 3CLpro, NSP15 and human COX-2 protein at 100 ns.

The Radius of gyration (rGyr) property was also examined to illustrate the stability of the tomatidine in the SARS CoV-2 3CLpro, NSP15 and human COX-2 binding pockets during the simulation of 100 ns (Figure 9). The rGyr parameter is used to measure how extended a ligand is, and is equivalent to its principal moment of inertia. The tomatidine in complexes with 3CLpro, NSP15 and human COX-2 exhibited an average rGyr value of 4.76 ± 0.036 Å, 4.77± 0.034 Å and 4.77± 0.034 Å, respectively (Table 1). No major fluctuation was observed in the rGyr. These constant values showed steady behavior. The post dynamic MM-GBSA analysis of free binding energy (ΔG Bind) calculation was carried out with the generation of 0-1001 frames having a 101-step sampling size. A total of 10 frames were processed and analyzed throughout the post dynamic MM-GBSA calculation of 100 ns MD data of tomatidine in complex with the SARS CoV-2 3CLpro, NSP15 and human COX-2 revealed by the dynamic’s studies. The computed post dynamic-MMGBSA based binding free energy for the protein ligand complexes is depicted in Table 1. The calculated average ΔG Bind of the complex tomatidine in complex with the SARS CoV-2 3CLpro, NSP15 and human COX-2 was found to be −47.4633 ± 9.28 kcal/mol, −51.8064 ± 8.91 kcal/mol and −54.8918 ± 7.55 kcal/mol, respectively. A more negative value shows stronger binding.

### 2.3. Pharmacokinetics, Drug-Likeliness and Toxicity Studies

A good drug must meet the conditions of absorption, distribution, metabolism and excretion (ADME) as well as toxicity and should entirely and quickly absorb from the gastrointestinal tract, specifically distributed to its target, metabolized in a manner that helps its activity, and excreted without any harm. Based on the predicted results summarized in Table 2, drug-likeness properties indicate that both tomatidine and patchouli alcohol obeyed to Lipinski’s, Veber’s and Egan’s rule assessing their flexibility as well as their surface area, with bioavailability score of 0.55 and consensus log Po/w of 4.90 and 3.57, respectively.

Lower hydrophobicity results in a decrease in the metabolism of compounds and high absorption. Pharmacokinetic data showed that both leads were permeators of the blood brain barrier and skin with high gastrointestinal absorption meaning their high absorbance potential. It is well known that cytochrome P450s can regulate the metabolism of various drugs. Tomatidine and patchouli alcohol were found to be no inhibitors of all cytochrome P450 isoforms CYP1A2, CYP2C19, CYP2C9 (except for PA), CYP2D6 and CYP3A4 which are the main players in Phase I metabolism. CYP3A4 as major enzyme system known to acts on lipophilic substrates and recognized to be responsible for the metabolism of about 60% of xenobiotics including drugs, carcinogens, steroids and eicosanoids. Both the leads exhibited negative results on Ames mutagenesis and therefore cannot be considered as a mutagenic agent. In addition, no toxicity was shown with the human ether-a-go-go- related gene (hERG I) inhibitor, and no hepatotoxicity and skin sensitization, which allows them to be good safety drugs.

The bioavailability radar (Table 2) of examined drugs represented by the pink area regrouping the optimal range for the followings (lipophilicity: XLOGP3 between −0.7 and +5.0, size: MW between 150 and 500 g/mol, polarity: TPSA between 20 and 130 Å, solubility: log S not higher than 6, saturation: fraction of carbons in the sp3 hybridization not less than 0.25, and flexibility: no more than 9 rotatable bonds with the colored zone defined the desired physicochemical space for good oral bioavailability suggesting that they fall entirely the pink area and therefore exhibiting good drug-likeness properties.

### 2.4. Target Prediction

**Figure 10:**
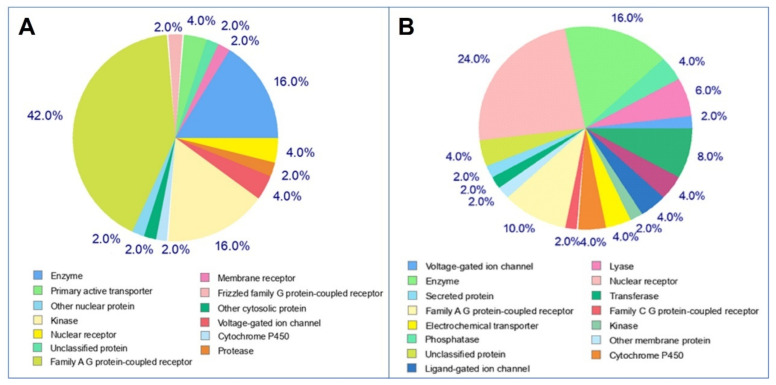
Pie-chart of top-50 of target predicted for selected molecules (**A**: tomatidine, **B**: patchouli alcohol).

To perform the molecular mechanisms underlying a given phenotype or bioactivity, and to rationalize possible side-effects, molecular target studies were predicted based on their resemblance with known drugs, to estimate their targets. The top 50 results of the closely associated receptors based on Target, Common Name, Uniprot ID, ChEMBL-ID, Target Class, Probability and Known actives in 2D/3D were illustrated as a pie-chart (Figure 10). As can be seen, tomatidine predicts 42% family AG coupled receptor, 16% enzyme, 16% kinase and 2% protease while, patchouli alcohol has 16% enzyme and 8% protease, as targets.

Overall, the obtained results highlighted the effectiveness of both tomatidine and patchouli alcohol as anti-SARS-CoV-2 agents targeting papain-like proteases (PLpro), 3C-like protease (Mpro) and NSP15 proteins. In fact, tomatidine has been demonstrated to inhibit the production of new Dengue virus particles [31]. More recently, Troost and colleagues [27] reported that this akkaloid exhibited antiviral activity against chikungunya virus by decreasing the number of infected cells and acted at a post-entry step of the virus replication cycle. It has also been elucidated that tomatidine inhibits porcine epidemic diarrhea virus (PEDV) by blocking 3CL protease activity in infected cells, and consequently PEDV replication [32]. Additionally, this molecule is active against Sunnhemp Rossette virus and Tobacco mosaic, and do not affect the replication of herpes simplex virus, human respiratory syncytial and influenza virus [33,34,35,36]. In 2021, Vergoten and Baily [37] studied the in silico interaction of four compounds including tomatidine, with Coronavirus 3C-like protease of PEDV (3CLpro) and (SARS-CoV-2)-main protease (Mpro). They reported that the calculated potential energy of interaction (ΔE) and free energy of hydration (ΔG) for the interaction of the two viral proteases (3CLpro and Mpro) with tomatidine were: −48.3, −21.6, −44.3, −19.1 (kcal/mol), respectively. Their results highlighted the potential inhibition of PEDV and SARS-CoV-2 main proteases by tomatidine. 

Similarly, patchouli alcohol was reported to possess antiviral activity against influenza virus [38,39] with an IC_50%_ about 2.635 μM. This tricyclic sesquiterpene was active against influenza A (H2N2) virus and especially after penetration of the virus into the cell, and strongly bind to neuraminidase protein with an interaction energy of −40.38 kcal mol⁻^1^ [40].

Many plant-derived molecules have been studied using computational approaches (docking and dynamic studies) targeting SARS-CoV-2 key enzymes including papain-like proteases (PLpro), 3C-like protease (Mpro) and NSP15 proteins. Several molecules have been reported to bind to active site of these three SARS-CoV-2 proteins with high binding affinity [41,42,43,44,45,46,47,48]. In silico studies have shown that various natural products have strong binding affinity to the non-structural proteins of SARS-CoV-2 virus (PLpro, Mpro and RdRp), and structural proteins such as spike (S) protein [43,49]. In fact, Teli and colleagues [45] described 60 plant-based natural compounds to combat COVID-19 virus interacting with SARS-CoV-2 (3C-like protease; PDB ID: 6LU7) and the high docking scores was about (−12.86 kcal/mol) for procyanidin A3, acetoside (−11.974 kcal/mol), rutin (−11.187 kcal/mol) and solanine (10.301 kcal/mol).

We also reported that emetine, a potent alkaloid isolated from the root of the Brazilian plant *Carapichea ipecacuanha*, was active against SARS-CoV-2 key enzymes using docking and dynamic studies [50]. Interestingly, this molecule showed significant binding affinity toward Nsp15 (−10.8 kcal/mol) followed by Nsp12 (−9.5 kcal/mol), RNA-dependent RNA polymerase, RdRp (−9.5 kcal/mol), Nsp16 (−9.4 kcal/mol), Nsp10 (−9.2 kcal/mol), Papain-like protein (−9.0 kcal/mol), Nsp13 (−9.0 kcal/mol), Nsp14 (−8.9 kcal/mol) and Spike Protein Receptor Domain (−8.8 kcal/mol) and chymotrypsin-like protease, 3CLpro (−8.5 kcal/mol), respectively.

## 3. Materials and Methods

### 3.1. Molecular Docking

Three-dimensional structure of the target proteins was retrieved from RCSB Protein Data Bank [51] with PDB ID: 6LUC, 6W9C, 6VWW, 5F1A and 4UY1 ligands-tomatidine and patchouli alcohol was selected for docking to identify the binding mode and binding affinity of the ligands in the binding pocket of the target protein [52]. 

Docking of protein ligands was carried out using AutoDock Vina [53], GOLD [54,55] and LibDock from Discovery Studio Client v20.1.0.19295 (DS) [Dassault Systemes, BIOVIA Corp., San Diego, CA, USA, v 20.1]. The docking softwares used employ different algorithms to improve binding accuracy. It has been demonstrated that these docking programmes are capable of predicting experimental poses with root mean squared deviations (RMSDs) that range between 1.5 and 2Å. Nonetheless, flexible receptor docking, particularly backbone flexibility in receptors, continues to pose a significant challenge to the currently available docking methods [56]. 

The binding affinity and gold fitness scores were obtained from AutoDock Vina and GOLD, respectively, for obtaining the best orientation and conformation of the ligands. To acquire accurate results, all the docking experiments were performed with the default parameters.

#### 3.1.1. Docking Using AutoDock Vina

PDBQT files of receptor protein and ligands were prepared using Graphical User Interface program AutoDock Tools (ADT). The bound ligand was removed, and the grid box was created around the ligand bind site of each of the protein. Dimensions of the grid around the binding site vary with each of the proteins. For 6LU7, the grid box was created with size 50 × 50 × 50 xyz points, grid spacing of 0.375 Å and grid center of x, y and z dimensions of −10.729, 12.418 and 68.816, respectively. For 6W9C, the grid box was set at 126 × 126 × 126 xyz points with grid spacing of 0.486 Å and grid center was designated at dimensions (x, y and z): −26.156, 34.011 and 25.506. For 6VWW, the grid box was created with size 58 × 64 × 66 xyz points, grid spacing of 0.375 Å and grid center was set at 72.309, 27.230 and −28.070 in x, y and z coordinates, respectively. For 5F1A, the grid box was created with size 52 × 62 × 60 xyz points with grid spacing of 0.375 Å and grid center was designated at dimensions (x, y and z): 30.725, 35.102 and 242.61o. While for 4UY1, the grid box was created with size 40 × 40 × 40 xyz points with grid spacing of 0.375 Å and grid center at −0.058, −2.230 and 0.718 in x, y and z coordinates, respectively. Protein and ligands were set to the rigid mode during the docking procedure and a configuration file consisting for protein and ligand information along with grid box properties was prepared for executing docking using AutoDock Vina. The ligand pose with highest binding energy/binding affinity was selected for exploring close intra-molecular interactions with the receptor.

#### 3.1.2. Docking Using GOLD (Genetic Optimization for Ligand Docking)

In GOLD suite, wizard was used for docking protein and ligands with default parameters. The active site was defined by selecting the bound ligand within the protein. 10 solutions for each ligand were obtained by applying default Genetic Algorithm settings. The best ligand was selected based on the highest GoldScore fitness function. The ligand and the protein docked complex was further analyzed for close intra-molecular interactions.

The molecular docking and visualization studies were also carried out with the help of commercially available site-features directed docking (LibDock) program in Discovery Studio. Each of the target protein was prepared and binding spheres was defined.. The docking preferences were set to “high quality”, and “Best” Conformation method with maximum conformations of 255 were selected. The ligand pose with highest LibDock Score was selected to form docked complex with the receptor for further analysis.

### 3.2. Intra-Molecular Interactions in Docked Complex

Docked complexes of the target proteins with tomatidine and patchouli alcohol obtained using AutoDock Vina, GOLD and LibDock were further analyzed for intra-molecular interactions using View Interaction tool from Discovery Studio Client v20.1.0.19295. Interacting residues of protein and ligand were visualized in 3D and 2D view.

### 3.3. Molecular Dynamic Simulation and Post Dynamic MMGBSA Binding free Energy Analysis

The molecular dynamics simulation was run using the “Academic version of Desmond” (Desmond 2020-3) to study the change in protein structure within the solvent system [57]. Desmond’s System Builder panel was used to design the water-soaked solvated system. For the simulations, the complex was centered in an orthorhombic cubic box with periodic boundary conditions and filled with single-point charge (SPC) water molecules buffered at a distance of a minimum of 10 Å between a protein atom and box edges [58,59]. The system was neutralized by adding counter-ions (Na^+^ and Cl^-^) at random, and an isosmotic state was maintained by adding 0.15 M NaCl. Using OPLS 2005 force field parameters as the default protocol associated with Desmond, the solvated built system was minimised and relaxed [60,61,62]. MD simulations were performed using an isothermal, isobaric ensemble (NPT) with a temperature of 300 K, pressure of 1 atm and a 200 ps thermostat relaxation period. A total of 100 ns simulations were run, during which 1000 trajectories were recorded. Finally, the Simulation Interaction Diagram (SID) tool was used to examine the MD simulation trajectory [63,64,65]. The interaction energies between the protein and the ligand poses were computed using the MM-GBSA (molecular merchandised generalized-born/surface area) method implemented by Schrodinger [23,24,25]. The average post dynamic binding free energy (ΔG bind) based on MM-GBSA was calculated using the thermal_mmgbsa.py script.

### 3.4. ADMET Prediction

Pharmacokinetic assessment of selected molecules was performed through the freely available online SwissADME web tool [66,67,68]. Toxicity predictions were assessed using the pkCSM server (http://biosig.unimelb.edu.au/pkcsm/prediction, accessed on 1 May 2021).

### 3.5. Molecular Target Predictions

Molecular target predictions are important to find the phenotypical side effects or potential cross reactivity caused by the action of small biomolecules were obtained by using the web tool (http://www.swisstargetprediction.ch/, accessed on 1 May 2021) and entering the smile formats of the desired drugs to obtain the targets. The prediction concerns the putative targets of the given molecule by utilizing 2D and 3D similarity index with known ligands.

## 4. Conclusions

In this study, we used docking-based virtual screening to identify potential inhibitors SARS-CoV-2 3CLpro, PLpro, NSP15, COX-2 and PLA2. Both tomatidine and patchouli alcohol modulate interactions that are required for infection by the SARS-CoV-2 virus. These two molecules possessed good pharmacokinetic and druglikness properties. In addition, Tomatidine was found to be potentially bind sufficiently to 3CLpro, NSP15 and human COX-2. To further validate the stability of a potential inhibitor tomatidine, a MD simulation study was performed for 100 ns. The MD simulation result revealed significant stability of test compound tomatidine in the active site of SARS-CoV-2 3CLpro, NSP15 and human COX-2, and exhibited good interactions with the surrounded amino acid residues. The post dynamic MM-GBSA analysis of the compound tomatidine showed significant binding affinity with its selected target. Moreover, much research is still required for the effectiveness of tomatidine in human clinical trials.

## Figures and Tables

**Table 1:** Radius of gyration of post dynamic-MMGBSA based Binding free energy (ΔG _Bind_) for the protein ligand complexes.

Complex Name	Radius of Gyration, rGyr (Å)		MMGBSA, ΔG _Bind_ (kcal/mol)	
	Mean	Range	Mean	Range
Tomatidine-3CLpro complex	4.76 ± 0.036	4.62 to 4.86	−47.4633 ± 9.28	−61.2963 to −31.8438
Tomatidine-NSP15 complex	4.77± 0.034	4.63 to 4.88	−51.8064 ± 8.91	−67.7417 to −35.2832
Tomatidine-human COX-2 complex	4.77± 0.034	4.63 to 4.86	−54.8918 ±7.55	−62.1318 to −38.2816

**Table 2:** Physicochemical properties, drug-likeness, bioavailability, pharmacokinetics and toxicity parameters of tomatidine (T) and patchouli alcohol (PA).

Physicochemical Properties			Drug-Likeness			Bioavailability (Radar Plot)			
	T	PA		T	PA	T			PA
**Molecular weight (g/mol)** **Num. heavy atoms** **Num. arom. heavy atoms** **Fraction Csp3** **Num. rotatable bonds** **Num. H-bond acceptors** **Num. H-bond donors** **Molar Refractivity** **TPSA (Å^2^)**	415.65 30 0 1.00 0 3 2 127.70 41.49	222.37 16 0 1.00 0 1 1 68.56 20.23	**Lipinski** **Ghose** **Veber** **Egan** **Muegge** **Bioavailability Score** **Consensus log P_o/w_**	Yes No Yes Yes No 0.55 4.90	Yes Yes Yes Yes No 0.55 3.57	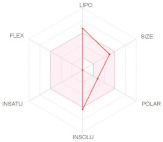			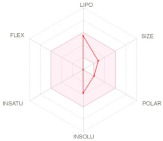
						LIPO: Lipophilicity FLEX: Flexibility SIZE: Size			INSAT: Insaturation INSOLU: Insolubility POLAR: Polarity
**Pharmacokinetics**									
	**T**	**PA**		**T**	**PA**			**T**	**PA**
**GI absorption**	High	High	**CYP1A2 inhibitor**	No	No	**CYP2D6 inhibitor**		No	No
**BBB permeant**	Yes	Yes	**CYP2C19 inhibitor**	No	No	**CYP3A4 inhibitor**		No	No
**P-gp substrate**	Yes	No	**CYP2C9 inhibitor**	No	Yes	**log Kp (skin permeation) cm/s**		−4.34	−4.87
**Toxicity**									
**AMES**			**hERG I inhibitor**			**Hepatotoxicity**		**Skin Sensitisation**	
**T**		**PA**	**T**		**PA**	**T**	**PA**	**T**	**PA**
No		No	No		No	No	No	No	No

## Data Availability

Not applicable.
